# Disseminated *Mycobacterium avium* complex infection mimicking malignancy in a patient with anti-IFN-γ autoantibodies: a case report

**DOI:** 10.1186/s12879-019-4564-4

**Published:** 2019-10-29

**Authors:** Yun-Kai Yeh, Jing-Ya Ding, Cheng-Lung Ku, Wei-Chih Chen

**Affiliations:** 1 Department of Chest Medicine, Taipei Medical University-Shuang Ho Hospital, Ministry of Health and Welfare, New Taipei City, Taiwan; 2 Department of Chest Medicine, Taipei Veterans General Hospital, Taipei, Taiwan; 3 Laboratory of Human Immunology and Infectious Disease, Graduate Institute of Clinical Medical Sciences, Chang Gung University, Taoyuan, Taiwan; 4 Department of Nephrology, Chang Gung Memorial Hospital, Taoyuan, Taiwan; 5 Institute of Emergency and Critical Care medicine and Faculty of Medicine, Medicine, School of Medicine, National Yang-Ming University, No. 201, Sec. 2, Shih-Pai Rd., Beitou District, Taipei, 11217 Taiwan

**Keywords:** Nontuberculous mycobacteria, Anti-interferon-γ autoantibodies, Sonography-guided biopsy, *Mycobacterium avium* complex

## Abstract

**Background:**

Disseminated nontuberculous mycobacteria (NTM) infections occur mostly in immunocompromised patients. Therefore, it is difficult to diagnose disseminated NTM infections in patients without history of immunocompromised diseases or using immunosuppressant. Patients with anti-interferon-γ (IFN-γ) autoantibodies are vulnerable to intracellular infections, such as disseminated NTM. Currently, there is no widely used and efficient technique for the detection of anti-IFN-γ autoantibodies. Herein, we report a case of an apparently healthy patient with disseminated *Mycobacterium avium* complex (MAC) infection who tested positive for anti-IFN-γ autoantibodies.

**Case presentation:**

A 64-year-old non-immunocompromised and apparently healthy Asian male presented to the emergency department with complaints of progressive chest pain for about 6 months and weight loss. A bulging tumour was found in the anterior chest wall. Chest computed tomography showed a lung mass over the right lower lobe and an osteolytic lesion with a soft tissue component at the sternum. Sonography-guided biopsies for the osteolytic lesion and sputum culture confirmed the presence of disseminated MAC infection. In addition, positive test result of anti-IFN-γ autoantibodies was noted. The patient was prescribed antibiotics. The lesions over the right lower lobe and sternum attenuated following the antibiotic treatment.

**Conclusion:**

Detection of anti-IFN-γ autoantibodies is important among previously healthy people with disseminated NTM infection. Presence of anti-IFN-γ autoantibodies may suggest a high risk of severe intracellular infection, such as disseminated NTM infection.

## Background

Nontuberculous mycobacteria (NTM) are a group of microorganisms ubiquitous in the environment. There are more than 160 species of NTM, of which at least 50 have been associated with pulmonary infectious disease. Unlike *Mycobacterium tuberculosis*, human-to-human transmission of NTM is not common. It is more difficult to treat NTM than *M. tuberculosis* due to drug resistance, and the treatment period usually lasts more than 12 months [1]. Although several studies have revealed NTM infections in both immunocompetent and immunocompromised patients, disseminated NTM infection is usually among immunocompromised patients such as people using long-term immunosuppressants or patients with human immunodeficiency virus (HIV) infection, particularly in those with CD4 counts below 50 cells/μL [2]. Interferon-γ (IFN-γ), which is secreted by natural killer (NK) cells and T cells, plays a critical role in cellular immunity. Previous studies have suggested that IFN-γ autoantibodies may play an important role in refractory and recurrent disseminated NTM infections [3]. Therefore, we present a case of a previously healthy patient with disseminated MAC infection who tested positive for anti-IFN-γ autoantibodies.

## Case presentation

A 64-year-old Asian male patient presented to the emergency department with complaints of progressive chest pain for about 6 months and weight loss. A review of the patient’s medical records revealed a prior history of benign prostatic hyperplasia and hypertension. There was no apparent history of alcohol consumption, smoking, illicit drugs herbs, or immunosuppressants.

**Fig. 1:**
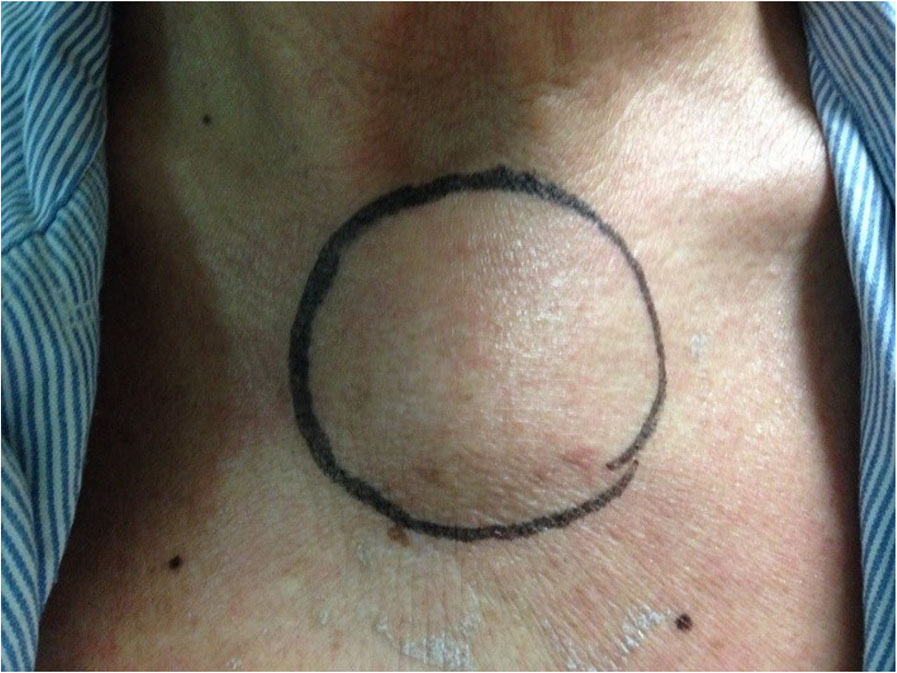
A bulging mass was found in the anterior chest wall

**Fig. 2:**
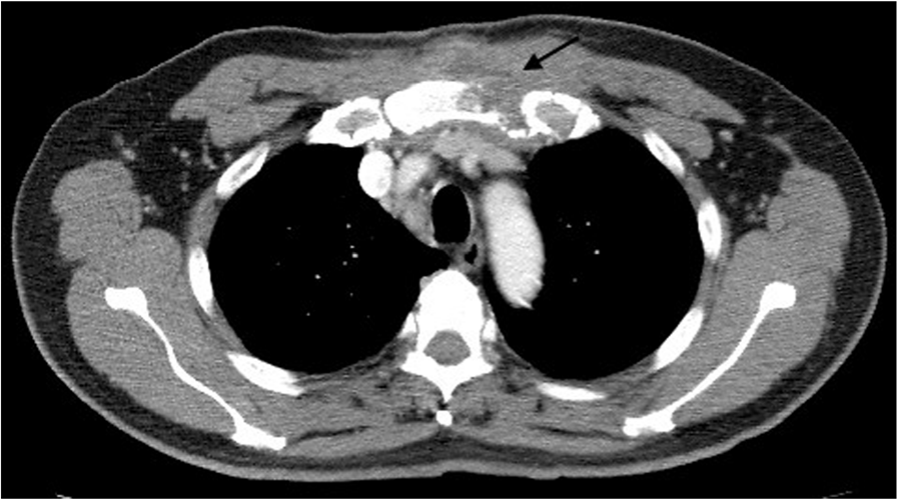
Arrow: Osteolytic lesion with a soft tissue component at the sternum

**Fig. 3:**
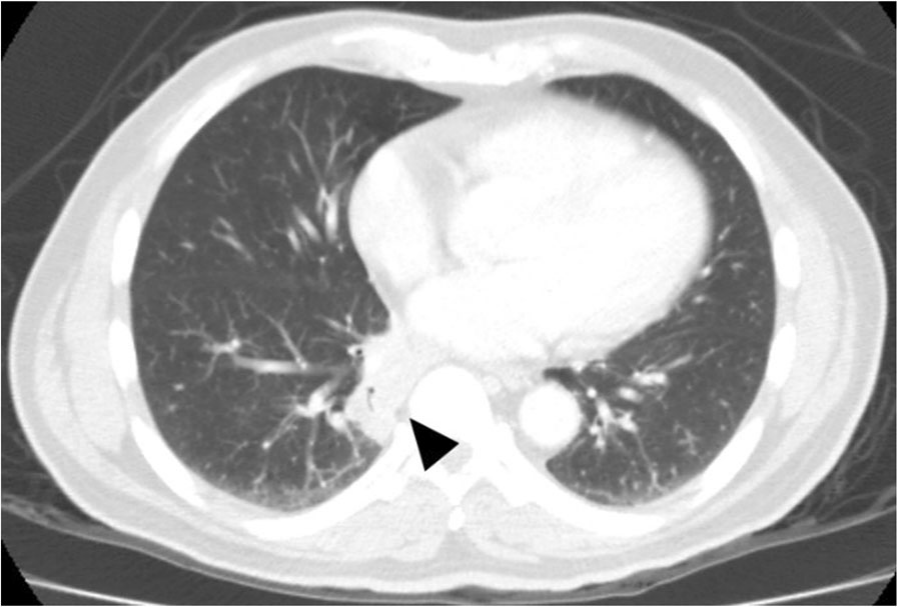
Arrowhead: Lung mass in the right lower lobe

**Fig. 4:**
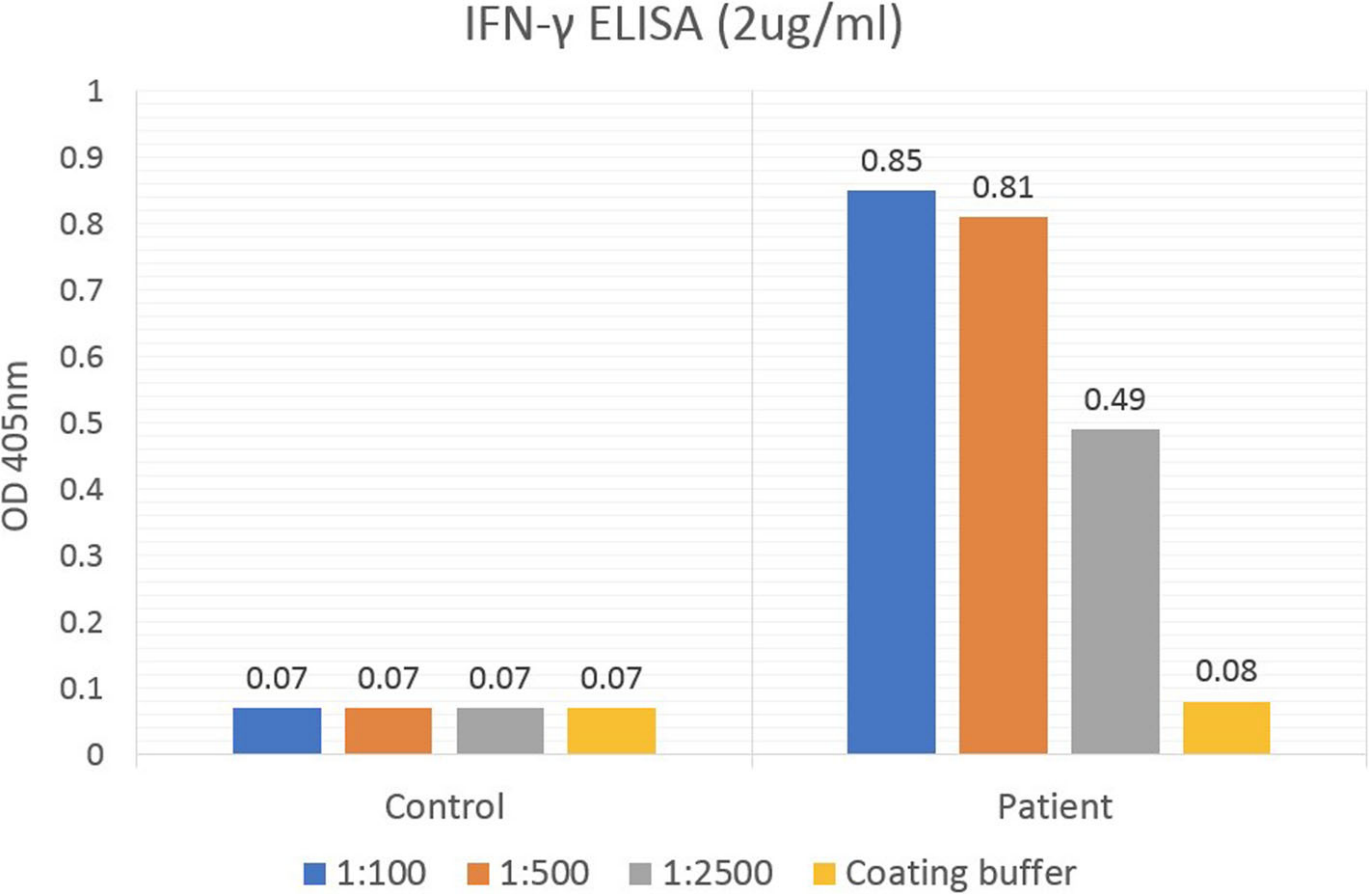
The presence of anti-IFN-γ autoantibodies measured by indirect ELISA. IFN-γ (2μg/ml, BD Bioscience) was coated in each wells; and serially diluted plasma samples from the patient and control samples (dilutions: 1:100, 1:500, and 1:2500) were added into wells. After washing, anti-IFN-γ autoantibodies were detected by anti-human IgG antibodies (The Jackson Laboratory) previous described [4]

A bulging mass was found in his anterior chest wall (Fig. 1). Notable laboratory findings included a white blood cell count of 11,400/μL and a C-reactive protein level of 8 mg/L. Chest computed tomography revealed an osteolytic lesion with a soft tissue component at the sternum mediastinal lymphadenopathy (Fig. 2, arrow), and a lung mass in the right lower lobe (RLL) (Fig. 3, arrowhead). Considering the possibility of lung cancer with mediastinal lymphadenopathy and bone metastasis, a sonography-guided biopsy was performed for the osteolytic lesion over the sternum. The pathological report indicated a focal granulomatous inflammation and necrosis without malignant cells. However, Ziehl-Neelsen staining was positive. Tissue culture and two sets of sputum all tested positive for *Mycobacterium avium* complex (MAC). Thus, MAC infection was suspected. Positive result of anti-IFN-γ autoantibodies was noted compared with control samples (Fig. 4). Although the patient did not report any prior history of immunosuppressant use and there was no serological evidence of HIV infection or autoimmune diseases, he was considered to be at risk of disseminated NTM infection due to a positive test result for anti-IFN-γ autoantibodies.

**Fig. 5:**
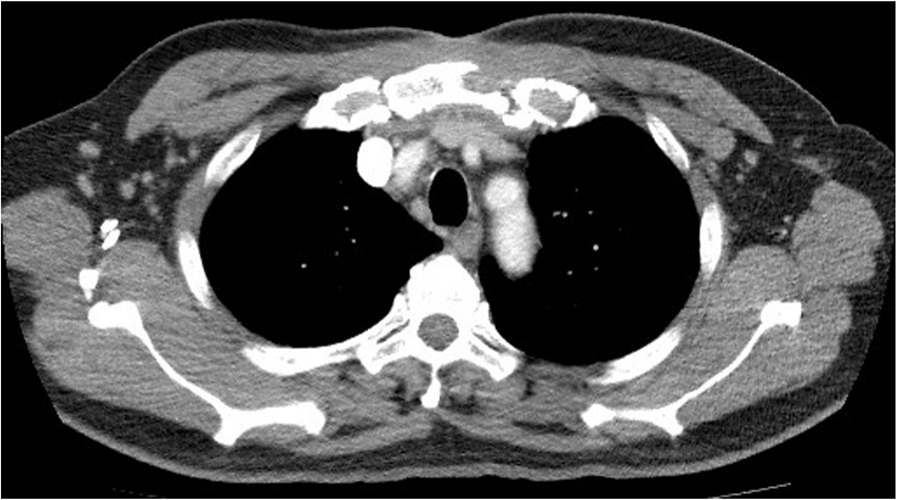
Osteolytic lesion at sternum diminished gradually after the treatment

**Fig. 6:**
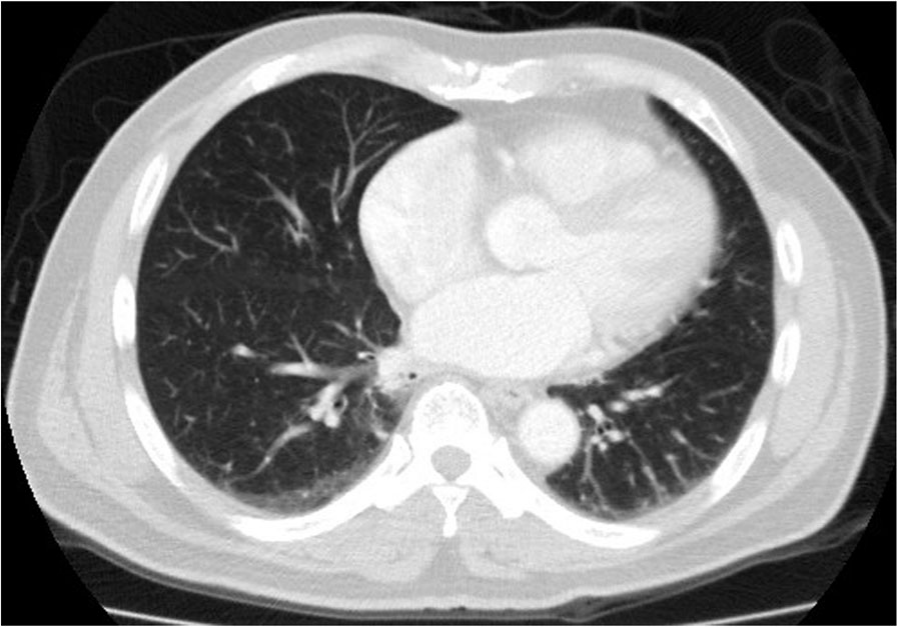
The size of osteolytic lesion decreased after the treatment

Hence, the patient was prescribed with oral form of clarithromycin 500 mg twice daily, oral form of rifabutin 300 mg daily, oral form of ethambutol 15 mg/kg daily, and intravenous amikacin 15 mg/kg three times a week for 3 months followed by oral form of clarithromycin 500 mg twice daily, oral form of rifabutin 300 mg daily, and oral form of ethambutol 300 mg daily for another 9 months. The lesions over the RLL and sternum diminished gradually following the treatment (Figs. 5 and 6).

## Discussion and conclusions

### Disseminated NTM

NTM are a group of microbes that are highly prevalent in the environment. Infections resulting from NTM have received more attention recently, especially among patients with chronic renal disease, malignancy, autoimmune disease, and acquired immune deficiency syndrome (AIDS). Nonetheless, the diagnosis of NTM infection can be challenging and biopsy is sometimes necessary to confirm the diagnosis of NTM infection [5]. Lymph nodes are the primary organs that are involved in an NTM infection, followed by osteoarticular system, bone, lung, and skin [4, 6]. Unlike *M. tuberculosis* (TB), NTM infection does not show human-to-human transmission. Moreover, NTM infection is more difficult to treat compared with infections due to TB because of the high level of resistance to available antimicrobial agents. For example, nearly all NTM species show resistance to pyrazinamide, which is one of mainstay anti-TB drugs. Furthermore, in the case of severe or disseminated NTM, the BTS guidelines recommend intravenous antibiotics, such as amikacin, for at least 3 months. Intravenous antibiotics using may lead to prolong hospitalization stay and influence treatment adherence [7]. Overall, the course of treatment for NTM infections usually lasts for more than 12 months. However, the severity and natural course of infections depend on the immunological status of the host. Long-term anti-NTM treatment maybe necessary among patient with incurable immunocompromised status. Disseminated MAC infections were very common in patients with advanced AIDS in developing countries before the widespread use of potent antiretroviral therapies [8]. Because disseminated NTM is common and life-threatening in people with advanced AIDS, mycobacterial antibiotic prophylaxis is recommended in patients with HIV infection and whose CD4 count is less than 50 cells/mm^3^ [9]. NTM infections occur mostly in immunocompromised patients. Hence, it is difficult to diagnosis disseminated NTM infection in non-immunocompromised patients. In our reported case, a patient without evidence of immunocompromised status was initially considered to have lung cancer instead of disseminated NTM infection.

### Anti-IFN-γ autoantibodies

Macrophages generate IL-12/23 that is stimulated by microbial components, such as lipopolysaccharides, and responses to intracellular infection. IFN-γ is synthesised by T cells and NK cells under IL-12/23 stimulation. In turn, IFN-γ can activate macrophages, which destroy intracellular microbes. IFN-γ/IL-12/23 axis plays a crucial role against intracellular infections, such as disseminated NTM infections, salmonellosis, other intramacrophagic bacterial infections, and reactivating latent varicella-zoster virus infection [3, 10]. Several studies have revealed the impact of IFN-γ/IL-12/23 axis defects. Kamijo et al. reported a study in which IFN-γ receptor-deficient mice were vaccinated with Bacillus Calmette-Guerin. Tumoral necrosis factor-α (TNF-α), IL-1α, and IL-6 could not be produced after infection in the IFN-receptor-deficient mice. Subsequently, these mice could neither form granulation nor kill mycobacteria because of cell-media immunity defects [11]. Mendelian susceptibility to mycobacterial diseases (MSMD) is a term used to describe a group of patients who are susceptible to mycobacterial diseases because of primary immunodeficiency diseases, including a defect of the interferon-γ receptor [12]. Anti-IFN-γ autoantibodies have received increasing attention recently and have been associated with disseminated, recurrence, and refractory NTM infections. Patients with high titer of anti-IFN-γ autoantibodies are vulnerable to intracellular microbes in a way similar to MSMD. High titer of anti-IFN-γ autoantibodies are defined as detectable anti-IFN-γ autoantibodies in plasma diluted 1:5000 [13, 14]. Regardless, the origin of anti-IFN-γ autoantibodies and its mechanisms remain unclear. Cases of patients with disseminated NTM because of anti-IFN-γ autoantibodies were first reported in 2004 [15, 16]. Patel et al. recruited 23 non-immunocompromised patients with disseminated NTM infection. Anti-IFN-γ autoantibodies were detected in 6 of the 23 patients (26%). All 6 patients with anti-IFN-γ autoantibodies were female, parous, and of Asian origin [13]. Another study by Chi et al. enrolled 17 Chinese adults diagnosed with disseminated NTM infection who otherwise did not have HIV infection, autoimmune diseases, diabetes mellitus, liver cirrhosis, or congenital immunodeficiency and were not undergoing immunosuppressive or immunomodulation therapy. All 17 (100%) patients tested positive for anti-IFN-γ autoantibodies, 35% had coinfection with salmonellosis, while 71% had herpes zoster infection [10, 17]. In the aforementioned studies, examination for anti-IFN-γ autoantibodies suggested disseminated NTM infection in the patients without known immunological defect, especially in those of Asian origin. Recent studies have shown that treatment with rituximab could improve the prognosis of disseminated NTM infection with anti-IFN-γ autoantibodies. Rituximab is a monoclonal antibody against the B cell protein CD20. It is recognised as a key cornerstone to treat diffuse large B-cell lymphoma. Rituximab may improve the recovery of IFN-γ signalling by IFN-γ-induced STAT1 phosphorylation [14]. Although large randomised controlled trials to support this hypothesis are lacking, rituximab could be an option or rescue treatment for disseminated NTM infection in patients with anti- IFN-γ autoantibody.

We presented a case of disseminated NTM infection in a previously healthy Asian male with anti- IFN-γ autoantibody. Detection of anti-IFN-γ autoantibodies may help to identify the risk of NTM infection, particularly in people of Asian origin. To date, there are no established differences in the treatments available for patients with or without anti-IFN-γ autoantibodies. Due to patient’s personal reasons, there was no serial data of titer of anti-IFN-γ autoantibodies in our case during treatment and clinical followup. However, disseminated NTM infection with anti-IFN-γ autoantibodies may indicate treatment failure and recurrent according to previous case reports. Rituximab may be considered as an optional treatment for refractory or recurrent NTM infection in patients with anti- IFN-γ autoantibody, although evidence to support this is currently lacking.

## Data Availability

The datasets used during the current study are available from the corresponding author on reasonable request.

## Abbreviations

AIDS: Acquired immune deficiency syndrome
BTS: British Thoracic Society
HIV: Human immunodeficiency virus
IFN-γ: Anti-interferon-γ
MAC: *Mycobacterium avium* complex
MSMD: Mendelian susceptibility to mycobacterial diseases
NK: Natural killer cells
NTM: Nontuberculous mycobacteria
RLL: Right lower lobe
TB: Tuberculosis
TNF-α: Tumoral necrosis factor-α
